# EphA5 knockdown enhances the invasion and migration ability of esophageal squamous cell carcinoma via epithelial-mesenchymal transition through activating Wnt/β-catenin pathway

**DOI:** 10.1186/s12935-020-1101-x

**Published:** 2020-01-13

**Authors:** Rui Zhang, Jing Liu, Wei Zhang, Lei Hua, Li-Ting Qian, Shao-Bing Zhou

**Affiliations:** 1 School of Clinical Medicine, Shan Dong University, Jinan, 250000 Shandong People’s Republic of China; 2 Department of Oncology, Qing Pu Branch of Zhongshan Hospital Affiliated to Fudan University, Shanghai, 201799 People’s Republic of China; 3 Department of Pathology, Taixing People’s Hospital, Taixing, 225400 Jiangsu People’s Republic of China; 4 Department of Provincial Clinical College, Anhui Provincial Hospital of Anhui Medical University, Hefei, 230031 Anhui People’s Republic of China; 5 Department of Radiation Oncology, The First Affiliated Hospital of USTC, Division of Life Sciences and Medicine, University of Science and Technology of China, Hefei, Anhui 230001 People’s Republic of China; 6 Department of Radiation Oncology, Taixing People’s Hospital, Taixing, Jiangsu 225400 People’s Republic of China

**Keywords:** Esophageal squamous cell carcinoma, EphA5, Epithelial-mesenchymal transition, Wnt/β‑catenin

## Abstract

**Background:**

The erythropoietin-producing hepatocellular (Eph) receptor A5 (EphA5) has been found to be overexpressed in some malignant tumors and is associated with disease prognosis. However, the role of EphA5 in esophageal squamous cell carcinoma (ESCC) is not clear.

**Methods:**

In the present study, we measured the expression of EphA5 in ESCC tissues and cell lines including KYSE150 and KYSE450 cells. siRNA transfection was used to interfere with EphA5 expression in ESCC cell lines. Cell viability, colony formation, scratch and invasion assays were performed to explore the roles of EphA5 in ESCC cell lines. Flow cytometry analysis was performed to investigate whether EphA5 could affect the cell apoptosis and cycle. The biomarkers related to epithelial-mesenchymal transition (EMT) and molecules associated with Wnt/β‑catenin signaling were also measured by western blot and immunofluorescence.

**Results:**

The protein and mRNA expression of EphA5 were significantly higher in fresh ESCC tissues and cell lines compared with normal control groups and human normal esophageal epithelial cells (HEEC). The cell viability assay and colony formation assay revealed that EphA5 knockdown enhanced the proliferation of KYSE150 and KYSE450 cells in vitro. The invasion and migration of ESCC cells were accelerated after EphA5 knockdown. The expression of EMT biomarkers was altered in ESCC cells transfected with siRNA targeting EphA5. Moreover, EphA5 downregulation enhanced the protein levels of β‑catenin and p-GSK-3β^Ser9^, which play a key role in the Wnt/β‑catenin pathway.

**Conclusions:**

EphA5 knockdown promotes the proliferation of esophageal squamous cell carcinoma,enhances invasion and migration ability via epithelial-mesenchymal transition through activating Wnt/β‑catenin pathway.

## Background

Esophageal cancer is one of the most common cancer and the sixth leading cause of cancer-related mortality and the seventh most prevalent cancer globally [1, 2]. There are two main subtypes: esophageal squamous cell carcinoma (ESCC) and esophageal adenocarcinoma (EAC). EAC is prevalent in developed countries, while ESCC is more frequent in Asia and other developing countries. In the past few decades, the development of multidisciplinary treatments has improved the overall survival of ESCC patients, but the 5-year survival rate is less than 20% [2].

Erythropoietin-producing human hepatocellular carcinoma receptors (Eph receptors) are the largest subgroup of the receptor tyrosine kinases family. It was first identified in human cancers,and has also been found to play important roles in pathology and physiology [3]. Depending on their ephrin ligands, Eph receptors are subdivided into EphA or EphB subfamilies [4]. Eph receptors were originally found to regulate embryogenesis, by fine-tuning cell adhesion, location, and migration, especially in the nervous system [5]. Recent studies have also shown that these receptors regulate cell adhesion, differentiation and stemness [5]. EphA5, a member of Eph receptors, has been implicated in various biological activities, including tumorigenesis and progression of different cancers [6–10], radioresistance in lung cancer [11] and sensitivity to trastuzumab in HER2-positive breast cancer [12]. However, the role of EphA5 in patients with ESCC remains unknown.

Therefore, this study aimed to investigate the roles of EphA5 in ESCC and the mechanism by which EphA5 regulates ESCC cells, its association with metastasis and prognosis of patients with esophageal cancer.

## Methods

### Patients and samples

Patients with ESCC (N = 52) who underwent surgery at Taixing People’s Hospital were enrolled in this study. All patients were confirmed by pathology as squamous cell carcinoma, and did not receive preoperative radiotherapy or chemotherapy. According to their dates of operation, patients were divided into 2 groups. Tumor tissue samples and paired normal tissues used for immunoblotting were obtained from 4 patients diagnosed with esophageal carcinoma between September 2018 and October 2018 (group 1). The resected samples were briefly stored in a fridge at − 80 °C for subsequent experiments. Tumor specimens used in the immunohistochemical analysis were consecutively harvested from 48 patients with ESCC from 2016 to 2018 (group 2). This study was performed with the approval of the Biomedical Research Ethics Committee, and all patients signed written informed consent.

### Cell lines and culture

Human esophageal carcinoma cell lines (KYSE30, KYSE 70, KYSE 140, KYSE150, KYSE 180, KYSE 410, KYSE450, KYSE510) and human normal esophageal epithelial cells (HEEC) were provided by the Chinese Academy of Cell Resource Center (Shanghai, China). Cell lines were cultured in RPMI-1640 medium (Invitrogen, USA) containing 10% FBS (Invitrogen, USA) at 37 °C in a humidified chamber with 5% CO_2_.

### siRNA and plasmid transfections

The human esophageal carcinoma cells (KYSE150, KYSE450) were grown in 60-mm plates at about 30% confluence and transfected with siRNA duplexes after incubation for 24 h, using the riboFECT CP transfection kit (Ribobio, Guangzhou, China). The EphA5-targeting and β-catenin-targeting siRNA oligonucleotides were synthesized by Ribobio, Guangzhou, China. The β-catenin-targeting siRNA sequences (si-β-catenin) designed previously were used [13]. For siRNA-mediated EphA5 (si-EphA5) silencing, the following sequences were used: 5′-GGAAAGACGTGTCATATTA-3′ (si-EphA5#1) and 5′-GGCAGAACATAGCCCACTA-3′ (si-EphA5#2), The sequence of negative controls (NC) was a non-targeting sequence. The human EphA5 expression plasmid (p-EphA5) was provided by GeneCopoeia company. KYSE150 cells after EphA5 knockdown were transfected with the EphA5 overexpression vector by Lipofectamine 3000 (Invitrogen, USA) according to manufacturer’s protocol.

### Real-time PCR

Total RNA from the fresh frozen tissues and the treated cells at the logarithmic phase was isolated with Trizol (Tiangen Biotech Co, Beijing, China). Then, cDNA was synthesized from the 1ug RNA using the HiScript II Q RT SuperMix (Vazyme Biotech Co, Nanjing, China) according to the manufacturer’s instructions. The AceQ qPCR SYBR Green Master Mix (Vazyme Biotech Co, Nanjing, China) was used for quantitative-PCR to detect the amount of EphA5 and β‑catenin mRNA, with Gapdh as the internal reference. The sequences of primers were:

- hEphA5-f:5′-TCTGTGGTACGACACTTGGC-3′;
- hEphA5-r:5′-CTTGCACATGCATTTCCCGA-3′;
- hβ‑catenin-f:5′-GCCAAGTGGGTGGTATAGAGG-3′;
- hβ‑catenin-r:5′-GCGGGACAAAGGGCAAGA-3′;
- hGapdh-f:5′-TCCATGACAACTTTGGTATCGTG-3′;
- hGapdh-r:5′-ACAGTCTTCTGGGTGGCAGTG-3′.

### Cell viability assay

The cells transfection with the EphA5-targeting siRNA and non-targeting sequence were seeded into 96-well plates (3000 cells/well). Cell viability was determined with the cell counting kit-8 (CCK-8) according to the manufacturer’s protocol. The absorbance values of each plate were measured at a wavelength of 450 nm with a microplate reader (Thermo Fisher Scientific, USA).

### Colony formation assay

The treated cells were plated in triplicate on 6-well plates (250 cells/well) and cultured at 37 °C for 7–14 days for colony formation. Next, methanol was utilized to treat the cells followed and 0.1% crystal violet utilized to stain the cells. Finally, the stained colonies containing at least 50 cells were counted.

### Flow cytometry analysis

To analyze cell apoptosis and cell cycle, the KYSE150 and KYSE450 cells were harvested after transfection with siRNA duplexes for 72 h. For cell apoptosis analysis, collected cells were incubated with Annexin V-FITC and propidium iodide (Beyotime, shanghai, China) for 15 min and then detected with flow cytometry (Beckman, USA). For cell cycle analysis, we fixed the collected cells using 70% ethanol overnight at 4 °C. After that, the samples were incubated with propidium iodide (Beyotime, Shanghai, China) at 37 °C for 30 min in the dark. Next, the cells were tested within 2 h by flow cytometry (Beckman, USA).

### Western blot assay

The total protein of cells at the logarithmic phase was extracted by a lysis buffer. BCA Protein Assay Kit (Beyotime, Shanghai, China) was used to measure the total protein concentration. The harvested samples were heated for 10 min at 100 °C for denaturation. About 30 μg proteins were separated by gel electrophoresis and transferred to a polyvinylidene difluoride membrane (0.45 μm, Millipore, Billerica, MA, USA). The PVDF membrane was blocked with skimmed milk and then incubated with primary antibodies overnight at 4 °C. After incubation with HRP goat anti-rabbit or goat anti-mouse IgG antibody (Proteintech, Wuhan, China), the protein signal was captured with autoradiography films. The E-Cadherin, p-GSK-3β^Ser9^ and β-catenin antibodies were all obtained from Cell Signaling Technology. GSK3β, N-Cadherin, c-Myc, CyclinD1 and Gapdh antibodies were purchased from Proteintech, while EphA5 was purchased from Invitrogen. Snail was purchased from R&D Systems.

### Wound-healing assay

The migration of the treated cells was evaluated by wound scratch assays. After siRNA transfection for 24 h, the cells were plated on 24-well plates. For re-expression of EphA5 in KYSE150 cells transfected with siRNA, transient transfection of plasmids were performed. After about 24 h when the cells covered 80–90% of the bottom area of the 24-well plates, scratch wounds were made using a sterile 10 µL pipette tip. The distance and area covered by migrated cells were recorded using a microscope (Olympus Corp, Tokyo, Japan) at 0 h, 12 h, 24 h and 48 h after scratching.

### Invasion assay

Invasion assays were conducted using transwell chambers (Corning Incorporated, Corning, USA) with a thin coating of matrigel (BD Biosciences, USA). The dilution ratio of matrigel is 1:10. After transfection with siRNA, 1.5 × 10^5^ cells were seeded on the upper chamber containing medium without serum. The lower chamber was filled with 500 µl medium containing 10% fetal bovine serum. After 48 h of incubation, the cells were removed from the incubator, fixed and then stained. The invasive cells were counted in at least five random fields at a magnification of 100 × (Olympus Corp, Tokyo, Japan).

### Immunofluorescence

KYSE150 and KYSE450 cells treated for 24 h were seeded on 24-well plates containing an aseptic coverslip in each plate. After 24 h in the incubator, the cells were fixed with 4% paraformaldehyde, penetrated by 0.5% Triton X-100 and blocked with 5% bovine serum albumin. Subsequently, the primary antibodies were used for incubation at 4 °C overnight. The Cy3 goat anti-rabbit IgG antibody (1:400, ABclonal, Wuhan, China) was added and incubated for 1 h and DAPI (Sigma, USA) used to strain the nuclei for 5 min. The Images were captured with a fluorescence microscope (Olympus Corp, Tokyo, Japan). The primary antibodies (E-cadherin, p-GSK-3β^Ser9^, β-catenin) were obtained from Cell Signaling Technology and antibody against Snail was purchased from Genetex.

### Immunohistochemistry

Briefly, 4 μm sections prepared from stored FFPE human esophageal tumor tissues and the paired normal tissues were incubated at 65 °Cfor 30 min. They were then deparaffinized, rehydrated, and quenched with 3% H_2_O_2_. EphA5 was examined as described previously with anti-human EphA5 monoclonal antibody (1:50, Invitrogen, USA). The extent of immunostaining was assessed based on the staining intensity and percentage of stained tumor cells [14]. The point representing the percentage of positive tumor cells was: 0, 0–5%; 1, 6–25%; 2, 26–50%; 3, 51–75%; and 4, 75–100%. The intensity was graded as: 0 (negative), 1 (weak), 2 (moderate), and 3 (strong). The two primary scores were multiplied to obtain the staining index (SI). The SI ≥ 3 was considered as EphA5-high expression tumors, those with 0 < SI < 3 were regarded as EphA5-low expression while others with SI = 0 regarded as EphA5-negative expression.

### Statistical analysis

All experimental data were analyzed using Prism 7.0 and IBM SPSS Statistics 20. The 2-tailed Student’s t test, a χ^2^ test or a one-way analysis of variance was used to measure statistical significance. P < 0.05 indicated a significant difference.

## Result

### EphA5 Expression is increased in ESCC cells and patients

**Fig. 1:**
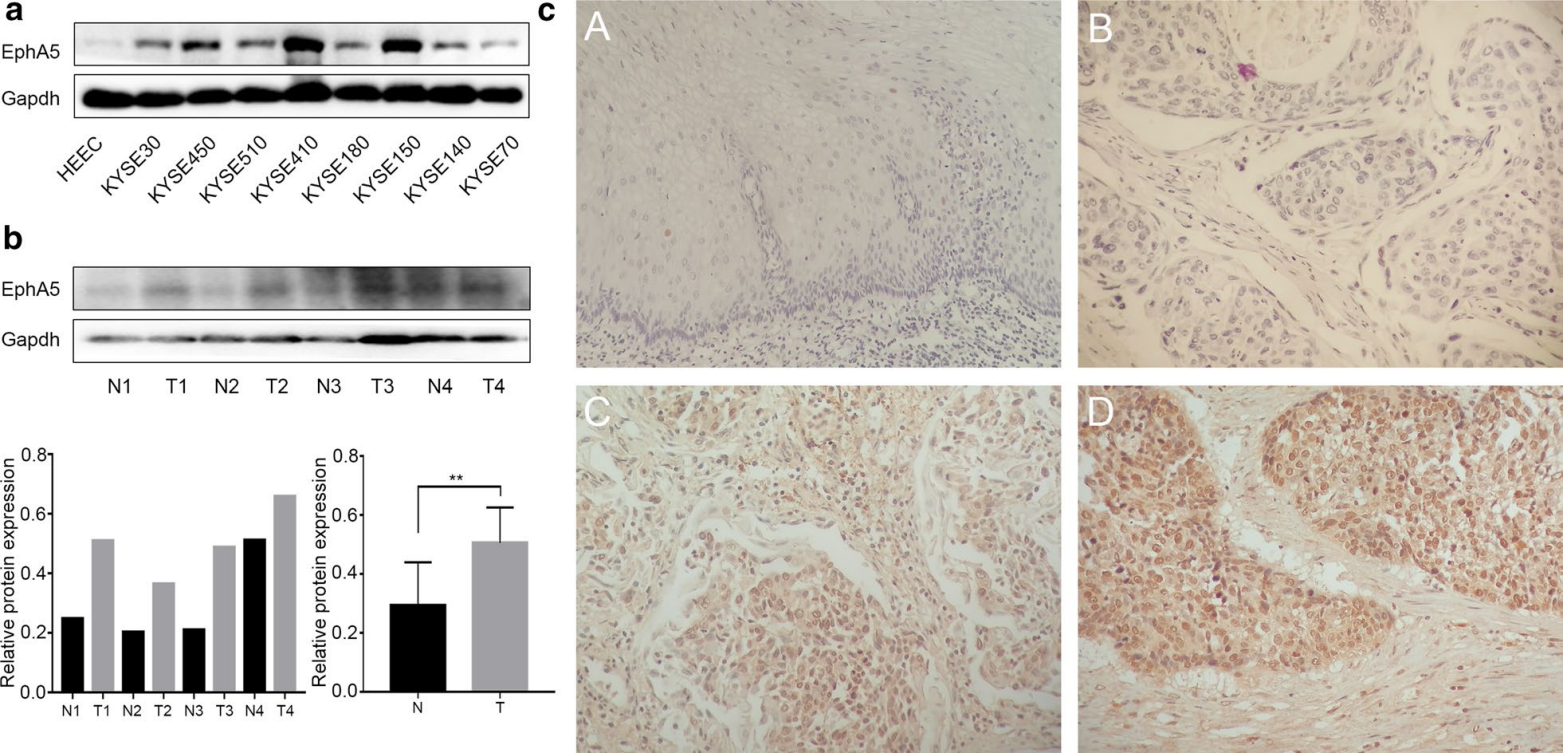
The expression of EphA5 in the ESCC cell lines and patients. **a** Western blot analyzed EphA5 expression in HEEC cells and ESCC cell lines KYSE30, KYSE450, KYSE510, KYSE 410, KYSE180, KYSE150, KYSE140, KYSE70. **b** The protein expression of EphA5 in 4 tumor tissue samples is higher than that in the paired normal tissues by western blot. **P < 0.01. **c** Representative images of EphA5 immunohistochemical staining in paracancerous tissues (**A**), and tumor tissues—EphA5-negative (**B**), EphA5-low (**C**), EphA5-high (**D**)

The protein expression of EphA5 in ESCC cell lines was analyzed by western blot assay (Fig. 1a). EphA5 was rarely expressed in HEEC cells, but was highly expressed in KYSE150, KYSE450 and KYSE410 cells. For KYSE150 and KYSE410 cells were from the patients with poorly-differentiated squamous cell carcinoma and KYSE450 cells was from the patient with well-differentiated squamous cell carcinoma, thus KYSE150 and KYSE450 cell lines were chosen for subsequently tests. Four fresh ESCC and normal samples were harvested during operation and preserved in − 80 °C refrigerator to be analyzed within 2 weeks. The EphA5 expression was detected by immunoblot analysis (Fig. 1b). Immunoblot analysis revealed that EphA5 expression of ESCC samples was higher than the normal samples, indicating that EphA5 may be involved in ESCC patients.

Subsequently, we analyzed EphA5 expression in 48 ESCC patients and 13 matched paracancerous tissues using archived paraffin-embedded tissues (Fig. 1c and Table 1). EphA5 was not detected in the squamous epithelium of the paracancerous tissues but was weakly expressed in the basal cells. No strong positive expression was detected in all patients, and all EphA5 SI were less than or equal to 6 points. There was no significant correlation of EphA5 with gender, age, TNM stage, vascular and neurological invasion. Among the 48 patients, regional lymph node metastasis occurred in 24 patients. In the 24 patients with positive lymph nodes, 8 patients had EphA5-high, whereas only 2 out of 24 patients with negative lymph nodes had EphA5-high. No statistical difference between the two groups was found, which may be due to the small number of samples. Among 10 ESCC patients with high expression of EphA5, there were 7 patients with advanced tumor stage (T3 and T4), 8 patients with positive lymph nodes, and 9 patients with moderately differentiated squamous cell carcinoma. There were 6 male patients and 4 female patients. Thus, we conclude that high EphA5 expression is correlated with regional lymph node status, advanced tumor stage and moderately differentiated squamous cell carcinoma. Further analysis showed that the EphA5 SI of patients with only neurological invasion but without vascular invasion (n = 4) was less than 2, implying that low EphA5 expression was correlated with neurological invasion.

**Table 1:** Association of EPHA5 expression with clinicopathological features in ESCC patients

Characteristic	Patients with FFPE	EPHA5 expression		χ^2^	*P* value^a^
	Tissue (n = 48)	Negative + low	High		
Age				0.844	0.358
≤ 65	23	20	3		
> 65	25	18	7		
Sex				0	1
Male	34	27	7		
Female	14	11	3		
Histologic grade				0.448	0.503
Grade I–II	37	28	9		
Grade III	11	10	1		
TNM stage				0.034	0.854
I-II	18	15	3		
III-IVA	30	23	7		
Lymph node status				3.158	0.076
Negative	24	22	2		
Positive	24	16	8		
Vascular or nerve invasion				0	1
Negative	34	27	7		
Positive	14	11	3		

Lymph node status: negative, no positive nodal metastases; positive, number of positive nodal metastases ≥ 1

FFPE, formalin fixed paraffin-embedded

^a^Pearson’s χ^2^ test

### EphA5 knockdown promoted the proliferation, migration, and invasion of ESCC cells

**Fig. 2:**
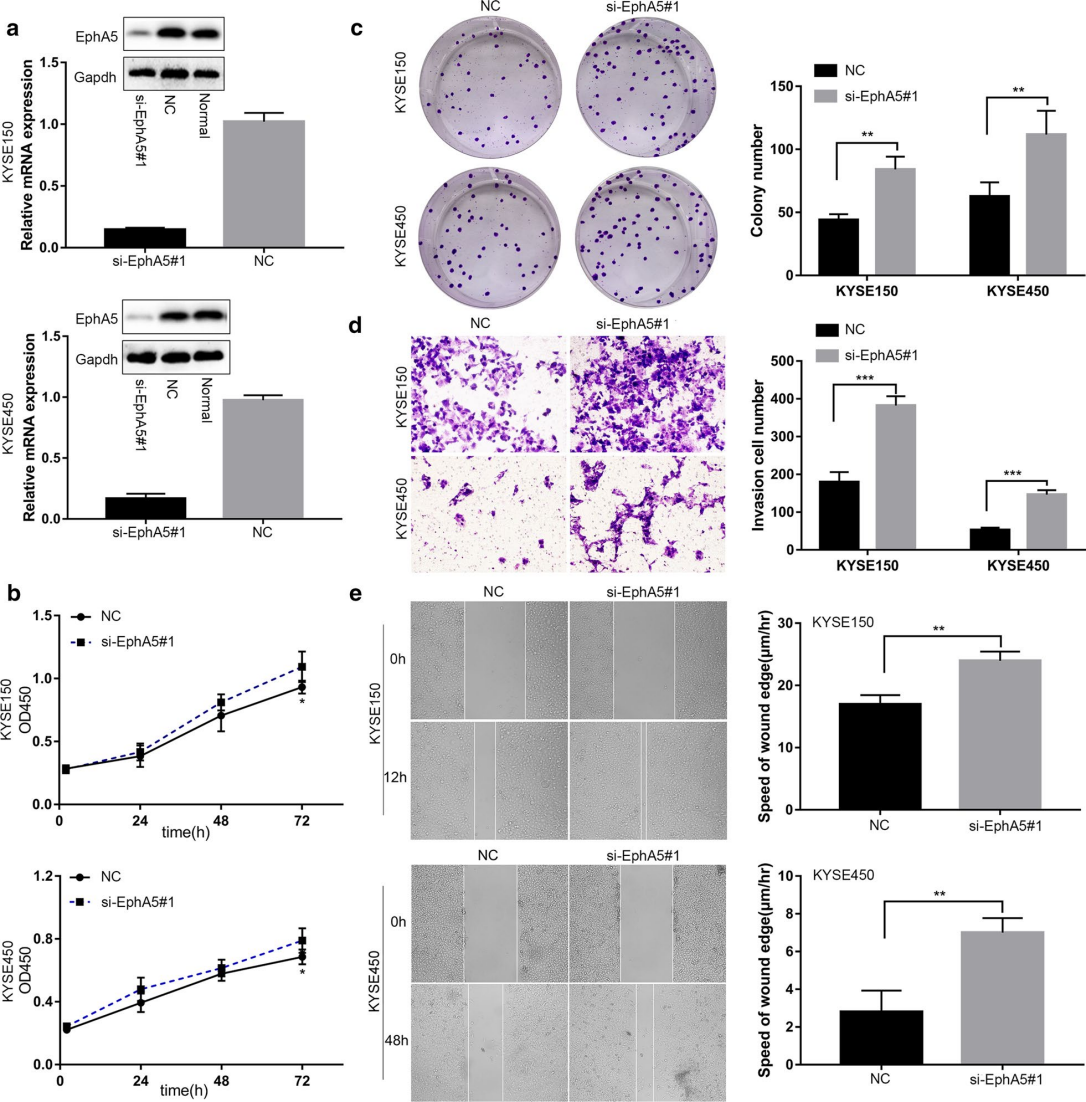
Knockdown of EphA5 promoted the proliferation, migration, and invasion of ESCC cells in vitro. **a** Western blotting and qRT-PCR results showed that EphA5 expression in KYSE150 and KYSE450 cells was downregulated by siRNA treatment. **b** The proliferation rate of the si-EphA5 groups was higher than that of the NC groups in KYSE150 and KYSE450 cells. **c** The colonies formed by cells treated with si-EphA5 was more than that of NC groups in KYSE150 and KYSE450 cells. **d** EphA5 knockdown significantly promoted the invasion of KYSE150 and KYSE450 cells compared with the NC groups. **e** Wound-healing assay showed knockdown of EphA5 enhanced cell migration in KYSE150 at 12 h and KYSE450 cells at 48 h. *P < 0.05, **P < 0.01, ***P < 0.001 versus NC group

To further explore the roles of EphA5, KYSE150 and KYSE450 cells were transfected with siRNA. This transfection decreased EphA5 protein and mRNA expression significantly (Fig. 2a, Additional file 1: Fig S1a, b). Next, we evaluated whether EphA5 could regulate the ESCC cells proliferation by the cell viability assay and colony formation assay. The cell viability assay showed that EphA5 knockdown accelerated the proliferation of KYSE150 cells and KYSE450 cells (Fig. 2b, Additional file 1: Fig. 1c). We observed that the number of colonies formed by cells with EphA5 knockdown was more than that of negative controls (Fig. 2c). Having shown that EphA5 knockdown enhanced the cell proliferation, we then analyzed the cell apoptosis and cell cycle by flow cytometry. Interestingly, there was no significant difference between the EphA5 knockdown cells and negative controls.

Further tests were performed to determine the effects of EphA5 on the migration and invasion of KYSE150 and KYSE450 cells. In the wound-healing assay, the cells with EphA5 knockdown displayed significantly accelerated wound healing compared to the respective controls (Fig. 2e, Additional file 2: Fig. S2a, b). Transwell invasion assay revealed that EphA5 knockdown significantly increased the invaded number of cells and promoted the invasion ability (Fig. 2d, Additional file 2: Fig. S2c). When EphA5 was re-expressed in the KYSE150 cells interfered with siRNA, the speed of wound healing was slowed down and the invaded number of cells was decreased compared with the KYSE150 cells with EphA5 knockdown (Additional files 3, 4: Figs. S3, S4).

### EphA5 knockdown induced EMT in ESCC

**Fig. 3:**
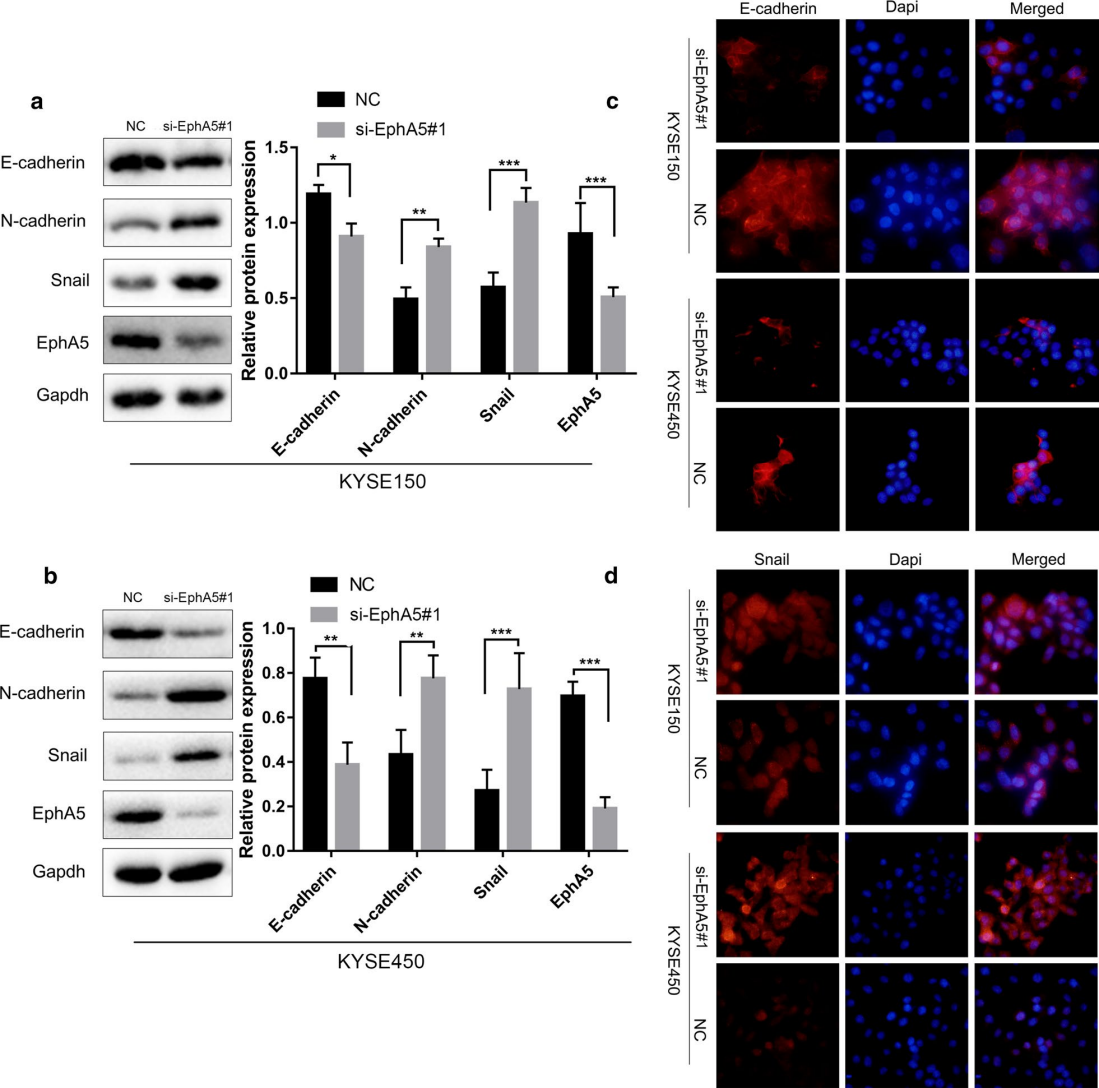
EphA5 knockdown promoted the occurrence of epithelial and mesenchymal. **a**, **b** Cells were analyzed by western blot to determine the protein expression of epithelial and mesenchymal related markers in KYSE150 and KYSE450 cells. **c**, **d** Expression of E‑cadherin and Snail was detected by immunofluorescence analysis. Nuclei were counterstained with DAPI (blue). *P < 0.05, **P < 0.01, ***P < 0.001 versus NC group

To explore whether EphA5 promoting the migration and invasion was associated with EMT process, we measured the protein levels of EMT markers (E-cadherin, N-cadherin, Snail) in the KYSE150 and KYSE450 cells after siRNA infection. As expected, E-cadherin was decreased, while N-cadherin and the relevant transcription factor Snail were remarkably increased in KYSE150 and KYSE450 cells with EphA5 knockdown compared with the controls cells (Fig. 3a, b). Immunofluorescence analysis was also performed to verify the alteration of E-cadherin and Snail expression (Fig. 3c, d). Consistently, silencing of EphA5 promoted EMT in KYSE150 and KYSE450 ESCC cells.

### EphA5 knockdown regulated EMT by activating Wnt/β‑catenin signaling pathway

**Fig. 4:**
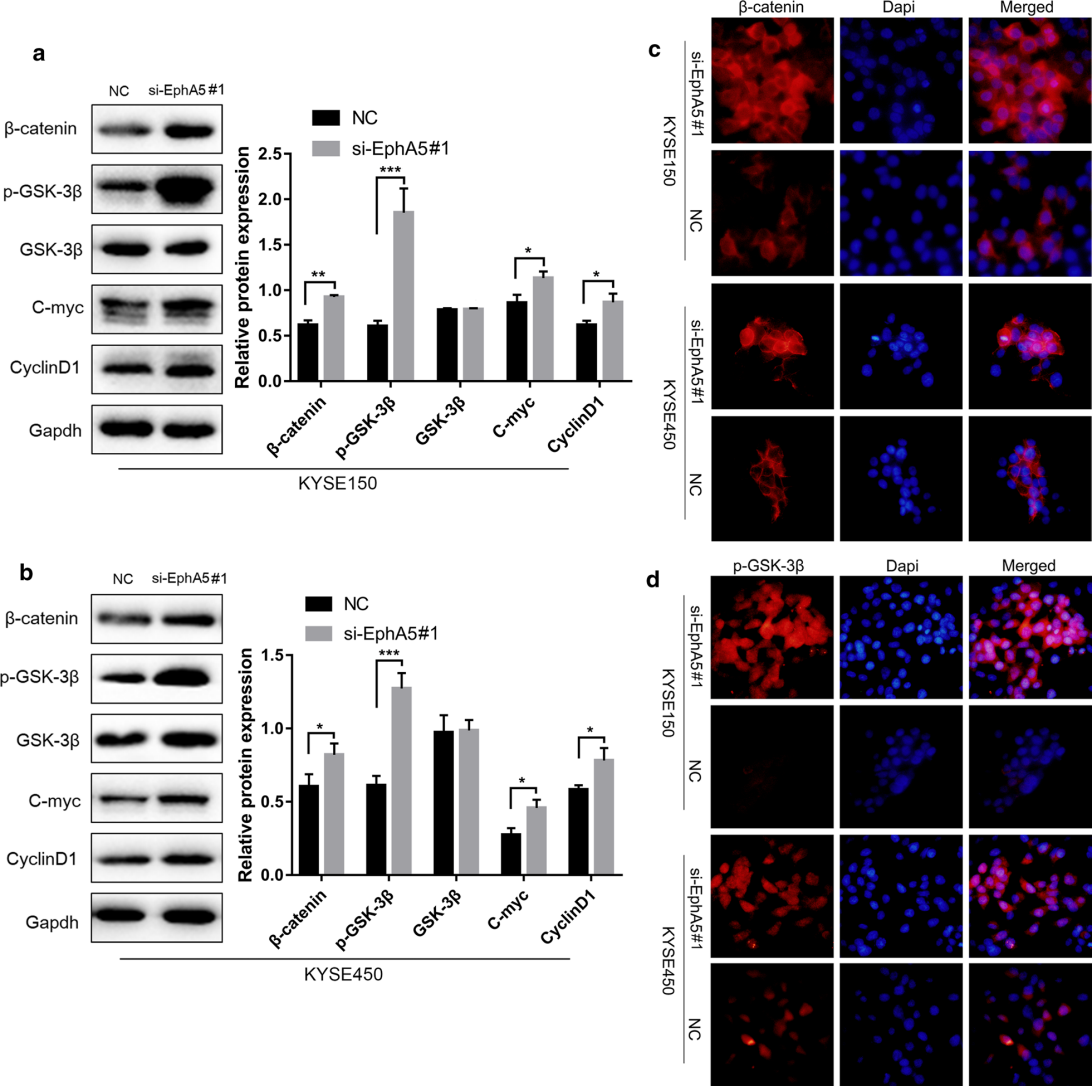
The effect of EphA5 knockdown on EMT was mediated by activating Wnt/β‑catenin signaling pathway. **a**, **b** Western blot was used to determine the expression of the key factors in Wnt/β‑catenin signaling (total and phosphorylated GSK-3β, β-catenin), and the downstream targets of Wnt/β‑catenin signaling(c-Myc and CyclinD1) in KYS150 and KYSE450 cells. **c**, **d** Immunofluorescence analysis showed β-catenin and phosphorylated GSK-3β localization and expression. Nuclei were counterstained with DAPI (blue). *P < 0.05, **P < 0.01, ***P < 0.001 versus NC group

**Fig. 5:**
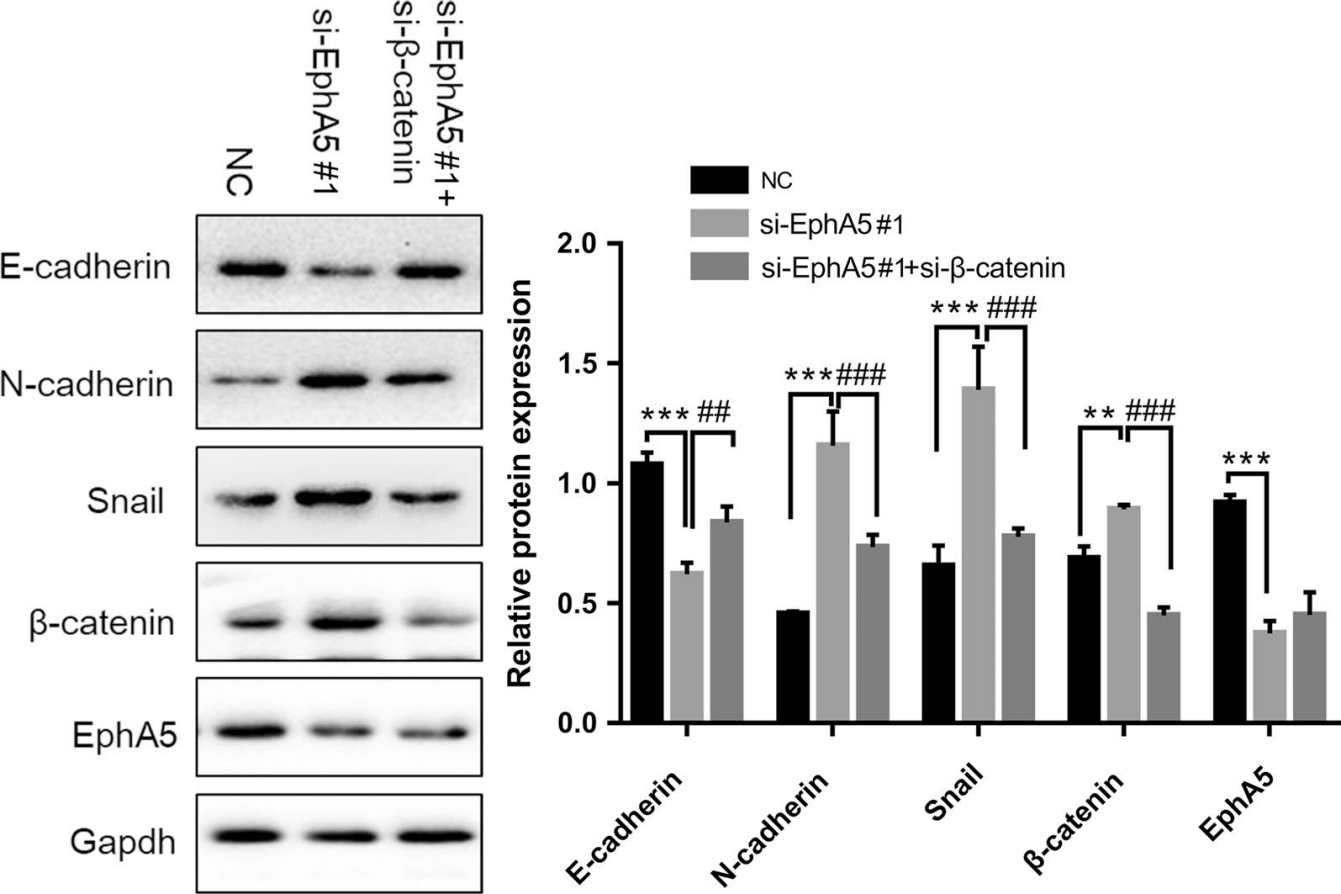
β‑catenin knockdown counteracted the effects of EphA5 on EMT. The expression of E‑cadherin, N‑cadherin, Snail, β‑catenin and EphA5 protein in KYSE450 cells transfected with si‑EphA5 and co-transfected with si‑EphA5 and si-β‑catenin was analyzed. **P < 0.01, ***P < 0.001 versus NC group; ^##^P < 0.01, ^###^P < 0.001 versus co-transfection group

To evaluate whether the effect of EphA5 knockdown on the EMT process was associated with Wnt/β‑catenin signaling pathway, the expression of total β-catenin, GSK-3β and phosphorylated GSK-3β (p-GSK-3β^Ser9^) were investigated. As shown in Fig. 4a, b, the expression of p-GSK-3β^Ser9^ was obviously increased by suppression of EphA5 together with the β-catenin compared with the controls by western blot. Similarly, immunofluorescence analysis showed increasing amounts of p-GSK-3β^Ser9^ expression concurrently with the increased β-catenin expression (Fig. 4c, d). Moreover, β-catenin expression could been observed in the cytoplasm and nucleus of cells with EphA5 knockdown, but it was only expressed on the cell membrane in the control groups (Fig. 4c, d). In addition, immunoblotting was performed to analyze the expression of c-Myc and CyclinD1, as the downstream targets of Wnt/β‑catenin signaling. As shown in Fig. 4a, b, EphA5 knockdown increased the expression of c-Myc and CyclinD1 compared with the negative controls. Therefore, whether the influence of EphA5 depletion on EMT was connected with the activation of Wnt/β‑catenin signaling should be further studied. For this reason, the KYSE450 cells were transfected with siRNA duplexes of both EphA5 and β-catenin. As shown in Fig. 5, the effect of cotransfection rescued the expression of E-cadherin and downregulated N-cadherin, Snail, and β-catenin expression compared with the cells only EphA5 knockdown. Thus, Wnt/β‑catenin signaling pathway was essential to the development of EMT triggered by EphA5 knockdown.

## Discussion

This current study shows that EphA5 is differentially expressed in esophageal cancer samples but not expressed in the squamous epithelium of the paracancerous normal tissues. We observed that EphA5 expression was high in 10 patients but low in 38 patients. Consistent with our study, Staquicini et al. [11] revealed that EphA5 was overexpressed in lung cancer while EphA5 was barely detectable in normal bronchial epithelium and alveoli. The study revealed that high EphA5 expression in lung cancer indicated higher locoregional recurrence and lower cumulative overall patient survival. But the association between EphA5 expression and regional lymph node metastasis or nerve and vascular invasion was not analyzed. And in high-grade hepatocellular carcinoma,the EphA5 gene was also demonstrated to be upregulated [15, 16]. However, other previous studies proved that EphA5 was detected to be low-expressed in some other malignant tumors [6–10]. The low expression of EphA5 was associated with lymph node metastasis of breast cancer [10], colorectal cancer [7] and gastric cancer [6], indicating poor prognosis in colorectal carcinoma [7] and ovarian cancer [8]. Contradictory with the above reports, we found that high EphA5 expression implies a greater likelihood of regional lymph node metastasis and advanced tumor stage, while low EphA5 expression may be related to nerve invasion. Additionally, increase of EphA5 expression was more often found in the ESCC patients with moderately differentiated squamous cell carcinoma. However, in ovarian serous carcinoma [8], loss of EphA5 expression was found to be associated with high-grade and advanced FIGO stage.

Next, we explored the roles of EphA5 and the associated molecular mechanisms in the ESCC cells. We found that EphA5 was expressed in all ESCC cells but was rarely expressed in HEEC cells. KYSE150, KYSE450 and KYSE410 cells exhibited higher EphA5 expression than other ESCC cells. KYSE150 cell line was derived from a 49 years old female patient with poorly differentiated squamous cell carcinoma from cervical esophagus, while KYSE450 cell line was from a 59 years old male patient with well differentiated squamous cell carcinoma from middle inhathoracic esophagus [17]. Thus KYSE150 and KYSE450cell lines were selected for further experiment. To downregulate EphA5 expression, we used siRNA duplexes to interfere KYSE150 and KYSE450 cells. Our finds showed that EphA5 knockdown enhanced malignant characteristics of KYSE150 and KYSE450 cells in vitro,such as the ability of cell proliferation, migration and invasion. It seems to be contradictory with the results that high EphA5 expression is related to lymph node metastasis in ESCC patients. To explain the contradictory data, an EphA5 overexpression plasmids was transfected into the EphA5 knockdown KYSE150 cells. We found that EphA5 overexpression could reverse the cancer-related characteristics in the KYSE150 cells with EphA5 knockdown. Thisis consistent with the report by Li et al. [9], which demonstrated EphA5 overexpression compressed the ability of prostate cancer cell migration and invasion. The possible reason is that EphA5 plays different roles in different tumors. Staquicini et al. [11] proved that after irradiation EphA5 silenced lung cancer cells displayed a defective G1/S cell cycle checkpoint. A recent study [12] reported that in HER2-positive breast cancers treated with trastuzumab EphA5 was involved in the cell cycle and apoptosis. EphA5 increased cell apoptosis and induced synthesis phase arrest in the presence of increasing concentrations of trastuzumab. However, the current study did not find cell cycle and apoptosis changes in the ESCC cells with EphA5 knockdown. As we known, cell cycle can be blocked by both irradiation and trastuzumab. Thence, we conclude that EphA5 is involved in the progress of cell cycle when external factors affect cell cycle. It has been shown that Epithelial-mesenchymal transition (EMT) is associated with various tumor functions including tumor cell migration, invasion, and metastasis [18–20]. Several researchers have shown that EMT plays an important role in ESCC [21–25] and is associated with the prognosis of patients [25]. Thus, the expression of E-cadherin, N-cadherin, and Snail was measured in ESCC cells following EphA5 knocked down. The results demonstrated that the level of E-cadherin in the EphA5 knockdown cells was downregulated whereas the levels of N-cadherin and Snail were upregulated compared with the negative controls. This indicated that EphA5 inhibition promoted migration and invasion by inducing EMT in ESCC. A recent study [12] also showed that EphA5 could regulated the expression of E-cadherin. Furthermore, the report [12] found that loss of EphA5 resulted in higher expression of cancer stem cell (CSC) markers in HER2-positive breast cancer cells, including CD44^+^/CD24^-/low^, NANOG, CD133+. So they concluded that EphA5 was involved in the Notch1 and PTEN/AKT signaling pathway.

It has been reported that EMT is regulated by multiple pathways including MAPK, Wnt, and PI3K pathways [18, 26, 27]. Previous researchers have found that in gastric cancer EMT process can be promoted by EphA2 through activating Wnt/β-catenin signaling [28, 29]. As EphA5 and EphA2 belong to the same family, thus the connection between EphA5 and Wnt/β-catenin signaling was evaluated. First, we found the p-GSK-3β^Ser9^ and β-catenin expression were markedly increased compared with the controls, which are very important in the classical Wnt/β-catenin signaling pathway. The levels of c-Myc and CyclinD1 were enhanced following EphA5 inhibition. And then to further confirm that the Wnt/β-catenin pathway linked EphA5 and EMT, β-catenin was depleted in the EphA5 knockdown cells by siRNA duplexes transfection. Western blot analysis showed that β-catenin depletion eliminated the effects of EphA5 knockdown on EMT, indicating a reversal of the EMT markers altered in the ESCC cells with EphA5 knockdown. As known, GSK-3β and β-catenin act key roles in the classic Wnt signaling pathway. When the activity of GSK-3β is inhibited by phosphorylation at Ser9 site, a decrease in the transcription coactivator β-catenin degradation results in β-catenin accumulating in the cytosol. β-catenin then translocates into the nucleus and binds to TCF factors, thereby promoting Wnt target genes expression, such as c-Myc and CyclinD1 [30].

The present study indicated that EphA5 knockdown increased the levels of N-cadherin and Snail, and yet decreased the E-cadherin expression. The p-GSK-3β^Ser9^ and β-catenin expression was also observed to be upregulated in the cells with EphA5 depletion, as well as c-Myc and CyclinD1. Together, these findings demonstrate that EphA5 knockdown can trigger EMT by activating Wnt/β-catenin signaling in ESCC.

## Conclusions

This study shows that EphA5 acts as an EMT suppressor through the Wnt/β-catenin pathway and hence plays an essential role in ESCC migration and invasion. EphA5 was highly expressed in ESCC tissues but lowly expressed in normal esophageal epithelial tissues. High EphA5 expression may promote lymph node metastasis, although this seems to be inconsistent with the in vitro results. Thus, whether the effect of EphA5 on other signaling pathways contributes to regional lymph node metastasis should be explored. And overexpression of EphA5 in ESCC cell lines need to be performed to better understand the roles of EphA5 in the future work. Studies enrolling more patients with esophageal cancer are required to examine the prognosis of ESCC patients. The direct regulatory mechanism of EphA5 in ESCC remains unknown and should also be explored in further studies.

## Supplementary information

**Additional file 1: Fig. S1.** Knockdown of EphA5 by si-EphA5#2 promoted the proliferation, migration, and invasion of ESCC cells in vitro. **a** Western blotting and qRT-PCR results showed that the protein(A) and mRNA(B) of EphA5 in KYSE150 cells was downregulated by siRNA treatment. **b** Western blotting and qRT-PCR results showed that the protein(A) and mRNA(B) of EphA5 in KYSE450 cells was downregulated by siRNA treatment. **c** The proliferation rate of the si-EphA5#2 groups was higher than that of the NC groups in KYSE150 and KYSE450 cells. *P < 0.05, ***P < 0.001 versus NC groups.

**Additional file 2: Fig. S2. a** Wound-healing assay showed knockdown of EphA5 by si-EphA5#2 enhanced cell migration in KYSE150(A) at 12 h and KYSE450(B) cells at 48 h. **b** EphA5 knockdown by si-EphA5#2 significantly promoted the invasion of KYSE150 and KYSE450 cells compared with the NC groups. **P < 0.01, ***P < 0.001 versus NC groups.

**Additional file 3: Fig. S3.** The expression of EphA5 in KYSE150 cells transfected with si‑EphA5 and co-transfected with si‑EphA5 and p-EphA5 was analyzed. **b** EphA5 knockdown significantly promoted the invasion of KYSE150 cells compared with the NC groups, while EphA5 re-expression could rescue the phenomenon. Representative images of the invasion cells—NC(A), si-EphA5 + p-EphA5(B), si-EphA5(C).*P < 0.05,**P < 0.01,***P < 0.001.versus si-EphA5 group.

**Additional file 4: Fig.S4.** Wound-healing assay showed knockdown of EphA5 enhanced cell migration in KYSE150 at 12 h, while EphA5 re-expression could rescue the phenomenon. ***P < 0.001.versus si-EphA5 group.

## Data Availability

All the data used for the present study is available from the corresponding authors on reasonable request.

## Abbreviations

EphA5: erythropoietin-producing hepatocellular receptor A5
ESCC: esophageal squamous cell carcinoma
siRNA: small interfering RNA
EMT: epithelial‑mesenchymal transition
HEEC: human normal esophageal epithelial cells
EAC: esophageal adenocarcinoma
