# Supplementary figures and images for: EphA5 knockdown enhances the invasion and migration ability of esophageal squamous cell carcinoma via epithelial-mesenchymal transition through activating Wnt/β-catenin pathway
Source: Cancer Cell Int. 2020 Jan 13;20:20. doi: 10.1186/s12935-020-1101-x (PMC6958788; doi:10.1186/s12935-020-1101-x)

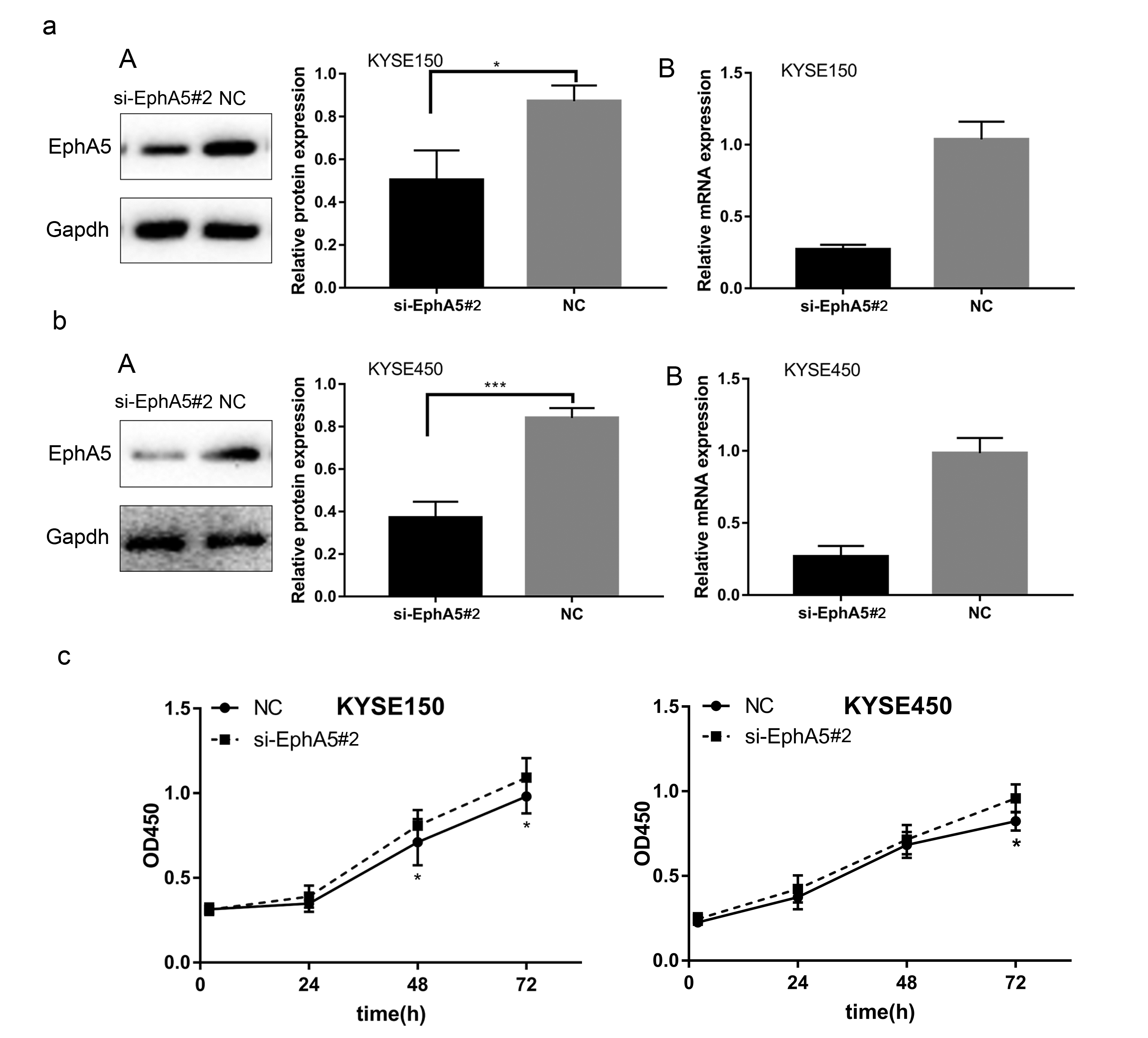

Supplement: Supplementary file 1 — Additional file 1: Fig. S1. Knockdown of EphA5 by si-EphA5#2 promoted the proliferation, migration, and invasion of ESCC cells in vitro. a Western blotting and qRT-PCR results showed that the protein(A) and mRNA(B) of EphA5 in KYSE150 cells was downregulated by siRNA treatment. b Western blotting and qRT-PCR results showed that the protein(A) and mRNA(B) of EphA5 in KYSE450 cells was downregulated by siRNA treatment. c The proliferation rate of the si-EphA5#2 groups was higher than that of the NC groups in KYSE150 and KYSE450 cells. *P < 0.05, ***P < 0.001 versus NC groups. [file 12935_2020_1101_MOESM1_ESM.tif]

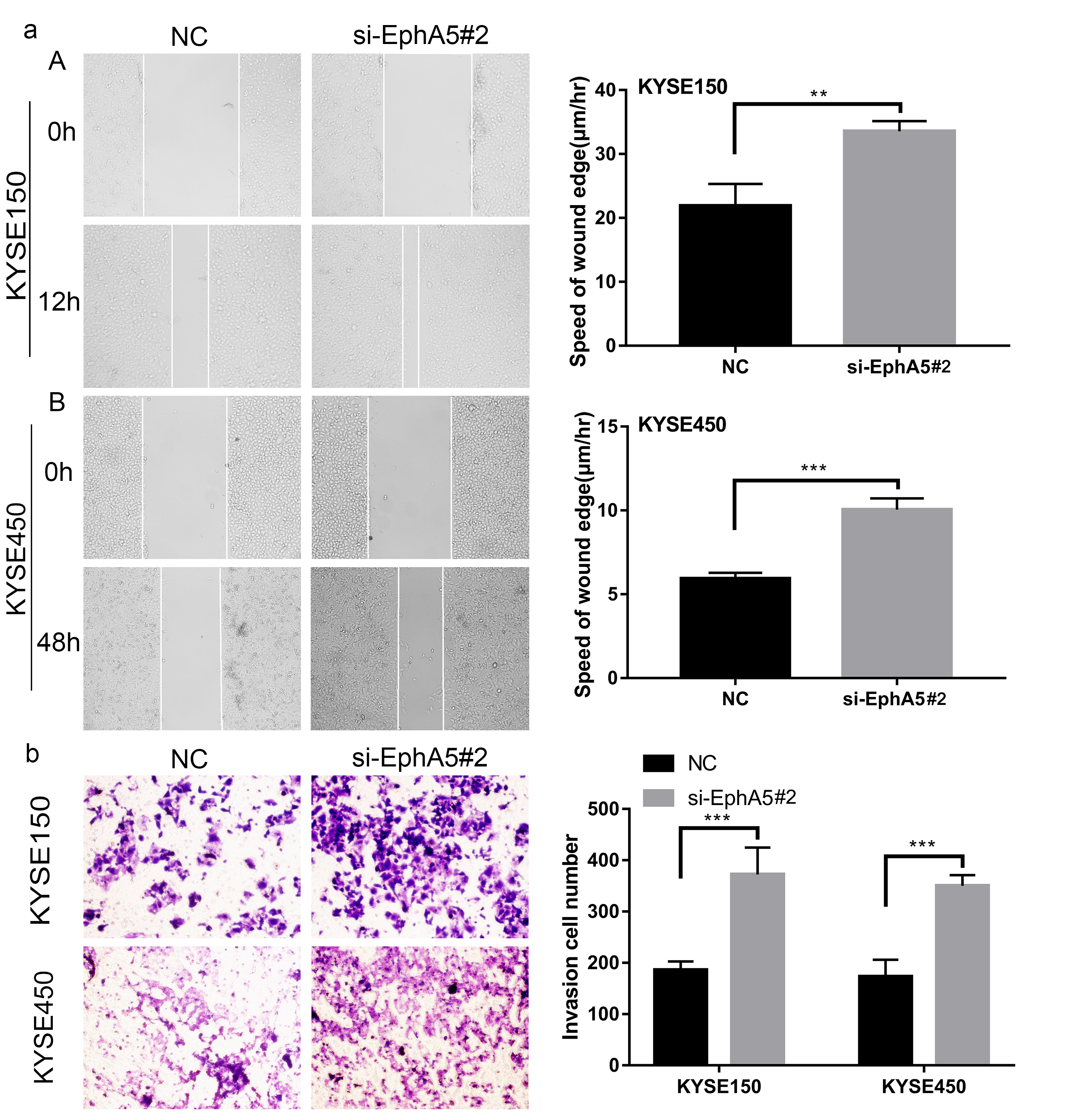

Supplement: Supplementary file 2 — Additional file 2: Fig. S2. a Wound-healing assay showed knockdown of EphA5 by si-EphA5#2 enhanced cell migration in KYSE150(A) at 12 h and KYSE450(B) cells at 48 h. b EphA5 knockdown by si-EphA5#2 significantly promoted the invasion of KYSE150 and KYSE450 cells compared with the NC groups. **P < 0.01, ***P < 0.001 versus NC groups. [file 12935_2020_1101_MOESM2_ESM.tif]

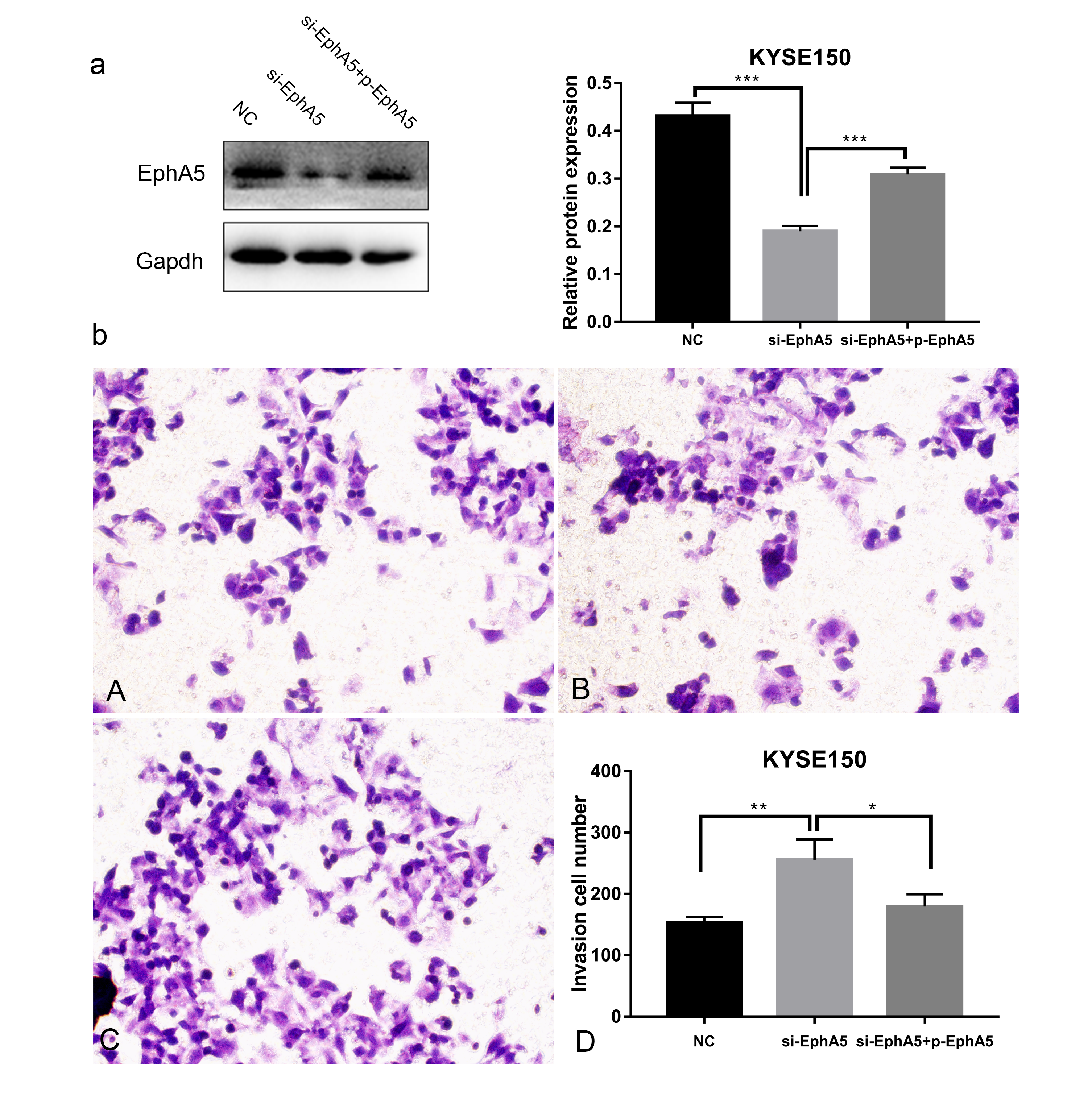

Supplement: Supplementary file 3 — Additional file 3: Fig. S3. The expression of EphA5 in KYSE150 cells transfected with si‑EphA5 and co-transfected with si‑EphA5 and p-EphA5 was analyzed. b EphA5 knockdown significantly promoted the invasion of KYSE150 cells compared with the NC groups, while EphA5 re-expression could rescue the phenomenon. Representative images of the invasion cells—NC(A), si-EphA5 + p-EphA5(B), si-EphA5(C).*P < 0.05,**P < 0.01,***P < 0.001.versus si-EphA5 group. [file 12935_2020_1101_MOESM3_ESM.tif]

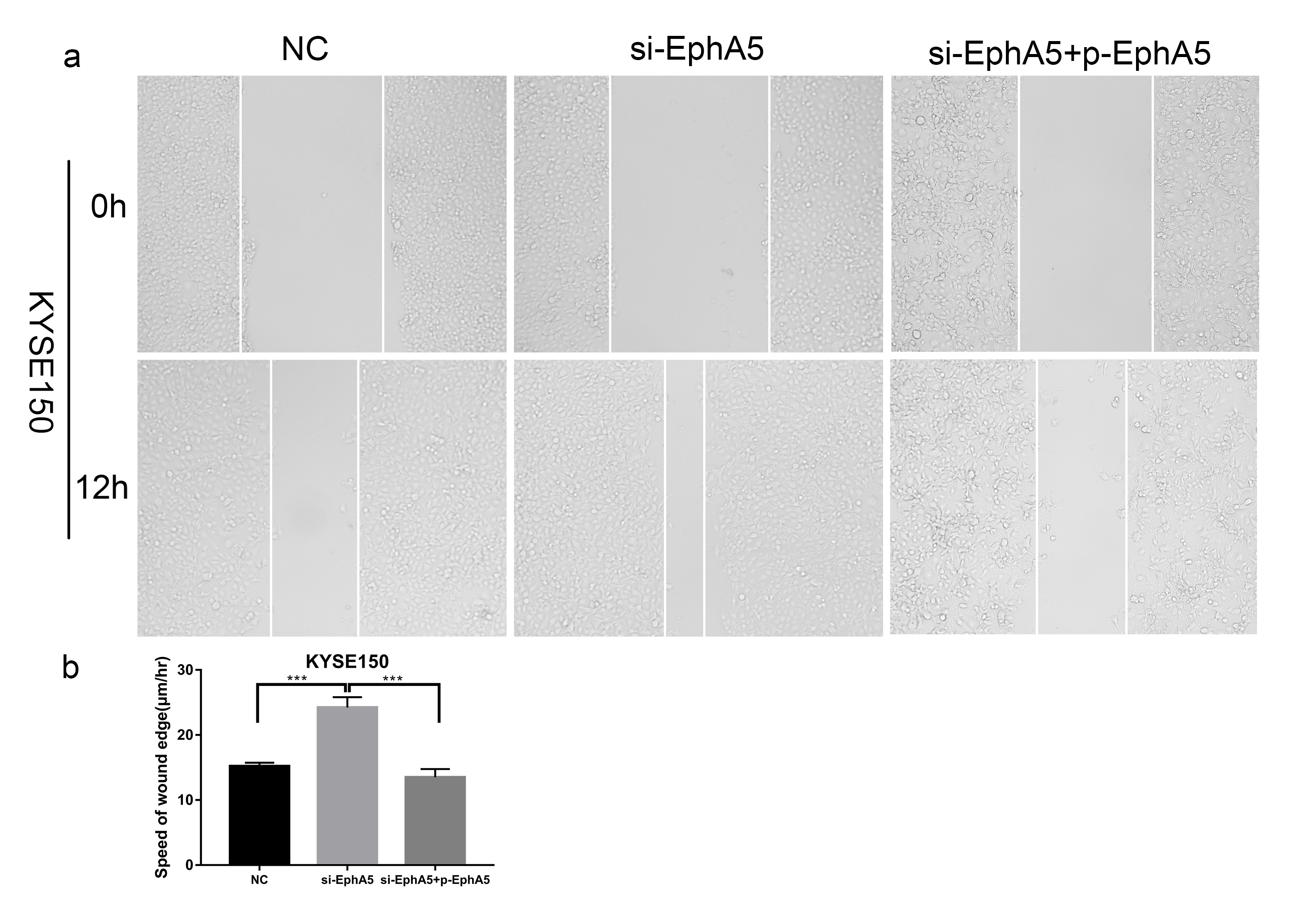

Supplement: Supplementary file 4 — Additional file 4: Fig.S4. Wound-healing assay showed knockdown of EphA5 enhanced cell migration in KYSE150 at 12 h, while EphA5 re-expression could rescue the phenomenon. ***P < 0.001.versus si-EphA5 group. [file 12935_2020_1101_MOESM4_ESM.tif]
